# Molecular Dynamics Investigation of Thickness Effects on Tensile Fracture and Component Migration in Asphalt Films

**DOI:** 10.3390/ma19091801

**Published:** 2026-04-28

**Authors:** Ruoyu Wang, Yanqing Zhao, Guozhi Fu, Yujing Wang, Qi Sun, Yin Zhao

**Affiliations:** 1 Department of Civil Engineering, Dalian University of Technology, Dalian 116024, China; 2 Department of Transportation and Logistics, Dalian University of Technology, Dalian 116024, China; 3 Department of Civil and Environmental Engineering, National University of Singapore, Singapore 117576, Singapore; 4 College of Water Resources and Architectural Engineering, Northwest A&F University, Yangling 712100, China; 5 Engineering School, Qinghai Institute of Technology, Xining 810000, China

**Keywords:** asphalt, tensile fracture, molecular dynamics, component migration, asphaltene index (I_A_)

## Abstract

Tensile fracture in asphalt involves complex mechanical responses and component migration. This study employs molecular dynamics (MD) simulations with the COPMASS II force field to investigate water intrusion at the asphalt–aggregate interface and subsequent tensile cracking at the nanoscale. To evaluate moisture damage, a ternary interface model was constructed using a specific distribution of water molecules at a target density. Results indicate that thickness significantly enhances moisture resistance; specifically, the asphalt film in the thinnest model (AS1) was penetrated by water molecules, leading to localized interfacial failure. Further uniaxial tensile simulations at a loading rate of 0.01 Å/psreveal that as film thickness increases (AS1 to AS4), the peak stress rises from 103.2 to 113.8 MPa, and the fracture energy increases from 136 to 747 kcal/mol. Based on the density redistribution of SARA fractions, component migration is divided into three stages: structural relaxation, resin-driven de-peptization, and polar component re-aggregation. Finally, the Asphaltene Index (I_A_) is proposed as a predictive indicator, showing that cracks consistently initiate in regions with minimum I_A_ values. These findings provide quantitative insights into the molecular mechanisms underlying asphalt durability.

## 1. Introduction

Cracks in asphalt pavements provide direct channels for moisture to infiltrate the internal structure [1]. Once moisture reaches the asphalt–aggregate interface, polar water molecules tend to displace the asphalt binder from the aggregate surface [2]. This process significantly weakens the interfacial bond and leads to debonding, a phenomenon known as moisture damage. Such damage eventually compromises the structural integrity of the pavement, triggering macro-scale distresses like surface raveling and pothole formation.

To study these mechanisms, researchers commonly use laboratory experiments to observe crack initiation and growth under controlled conditions. The semi-circular bending (SCB) test is widely utilized for its consistent results [3], while digital image correlation (DIC) techniques allow for the direct visualization of crack evolution [4]. Despite their widespread use, these traditional experimental methods have clear limitations. They often suffer from scale effects and high testing costs, making it difficult to fully replicate the behavior of actual in-service pavements.

Numerical simulations offer a viable method for investigating fracture behavior. Finite element analysis (FEA), specifically through XFEM or cohesive zone models (CZM), can accurately capture stress–strain distributions in fracture regions [5]. Similarly, the discrete element method (DEM) is effective for simulating mesoscale cracking by representing materials as particle assemblies based on CT scan data [6]. However, the reliability of these numerical models depends heavily on precise parameter calibration and significant computational resources.

Research on asphalt has recently shifted toward the microscale to gain fundamental insights. Atomic force microscopy (AFM) is now a primary tool in this field. It is used to observe water penetration [7], analyze the rheological impacts of asphalt fractions [8,9], and reveal internal morphology changes under loading [10]. Although AFM provides high-resolution surface data, molecular dynamics (MD) simulations have become a key method for quantitative analysis. Unlike experiments, MD simulations capture the structural evolution of asphalt through several key metrics:

(1) Radial Distribution Function (g(r)): This characterizes the spatial arrangement and density of asphalt molecules near the aggregate surface [11].
(2) Mean Square Displacement (MSD): This quantifies molecular mobility and water invasion rates. Studies show that water molecules can create cavities and weaken interfacial bonds.
(3) Relative Concentration Profiles: This analyzes the local distribution of molecules in the “sandwich” model. It explains how thickness affects interfacial resistance based on the specific number of molecules, rather than macro-scale assumptions like polymer entanglement [12,13].
(4) Interaction Energy: This provides a thermodynamic basis for debonding. MD results suggest that Coulomb interactions often dominate the work of adhesion, especially for alkaline minerals.

These molecular metrics help researchers understand the coordinated response of asphalt components during cracking. Recent MD studies have explored water intrusion [14,15,16,17] and microscale fracture [18,19,20,21]. For example, simulations can quantify peak stress and fracture energy. They show that failure modes transition from adhesive to cohesive as the loading rate decreases [22,23,24]. MD also evaluates how additives influence fracture resistance at the molecular level [18]. However, most MD studies treat asphalt as a bulk phase or use a single, fixed-thickness interface. They often overlook how film thickness and component distribution jointly influence the fracture path. At the nanoscale, film thickness is a decisive parameter. It determines the density and spatial arrangement of molecules. As thickness varies, the specific number of molecules belonging to different asphalt fractions changes significantly. These molecular changes and fraction migrations lead to shifting failure modes that macro-scale models cannot capture.

In this study, MD simulations were first used to model the intrusion of water molecules at the asphalt–aggregate interface, where interfacial fracture phenomena were observed. The effect of interface thickness on water-induced asphalt debonding was also investigated. The analysis was then extended to simulate the tensile fracture behavior of asphalt films with different thicknesses to clarify the influence of thickness on the tensile resistance of asphalt materials. Given the inherent heterogeneity of asphalt components, significant variations in mechanical behavior are expected in different regions. Based on the compositional characteristics of fracture-prone zones, a quantitative index is proposed to evaluate fracture susceptibility. This index may serve as a predictive tool for identifying potential crack initiation sites in asphalt materials.

## 2. Models and Methods

### 2.1. Construction of Asphalt Models with Different Thicknesses

The Molecular model adopted in this study employs an explicit-atom (all-atom) representation based on the COMPASS II force field, which is specifically optimized for high-accuracy predictions of the structural, cohesive, and thermophysical properties of organic asphalt components and mineral surfaces. A cut-off distance of 12.5 Å was employed for the simulation interactions. In addition, a time step of 1.0 fs was used for all molecular dynamics (MD) stages, including both equilibration and production runs. To effectively eliminate edge effects and simulate a continuous interface, periodic boundary conditions (PBC) were applied in all three Cartesian directions (x, y, and z).

**Figure 1:**
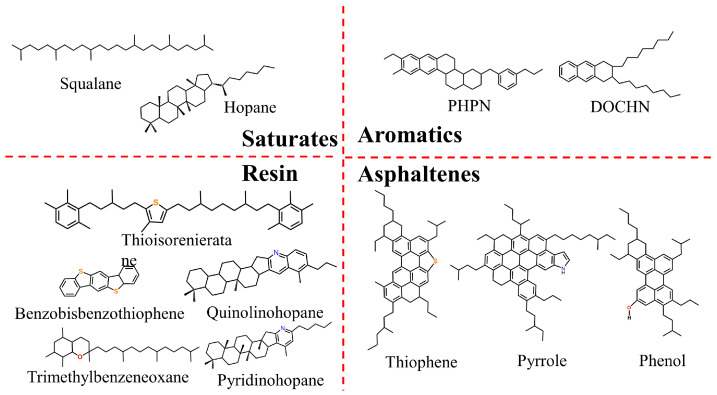
Schematic diagram of the asphalt model.

This research employs the 12-component asphalt molecular model developed by Li and Greenfield [25]. The rationale for selecting these specific molecules is based on the elemental compositions (mass fractions of C, H, N, S, O, and H/C atomic ratios) and the SARA fraction solubility classifications (Saturates, Naphthene Aromatics, Polar Aromatics, and Asphaltenes) provided in the SHRP database. Additionally, the model incorporates alkane size distributions measured by high-temperature gas chromatography. As shown in Figure 1, this 12-component model accurately represents the chemical diversity of real asphalt systems. The model has been validated to be in close agreement with experimental values regarding elemental composition, solubility distribution, and material density. Due to its reliability, this model is widely recognized in academia, and numerous scholars [13,15,26,27] have demonstrated its advantages in predicting the physical and rheological properties of asphalt.

**Figure 2:**
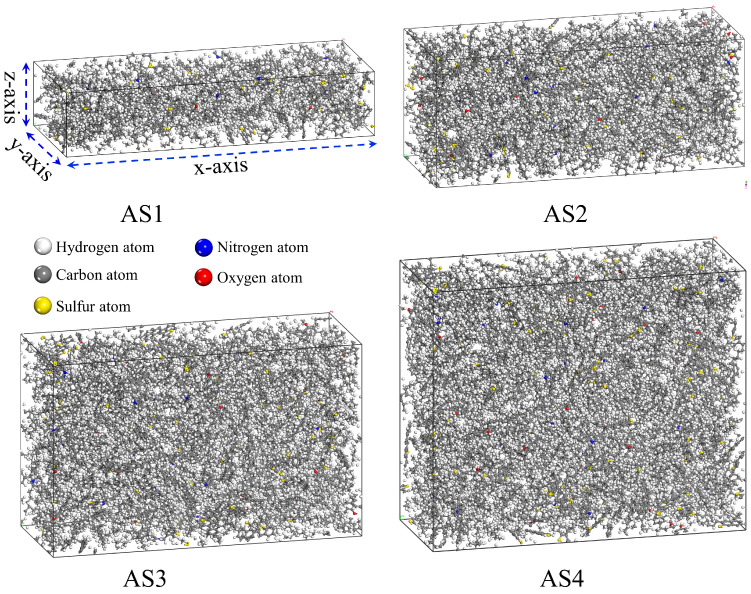
Schematic of asphalt layers with different thicknesses.

In this study, molecular models of asphalt films with different thicknesses were constructed using the Amorphous Cell module in Materials Studio, as shown in Figure 2. Based on the molecular composition ratios listed in Table 1, four asphalt models were developed with identical dimensions in the (x)- and (y)-directions but different thicknesses in the (z)-direction. The thickness variation was achieved by proportionally increasing the number of molecules according to the minimum base ratio while keeping the component proportions unchanged. Specifically, AS1, AS2, AS3, and AS4 contained 1, 2, 3, and 4 times the base number of molecules, respectively, thereby forming asphalt layers with progressively increasing thickness from the thinnest model (AS1) to the thickest model (AS4), as presented in Table 1. It is important to note that in our fully periodic systems, the term “thickness” specifically refers to the characteristic physical scale of the simulation cell along the (z)-axis. Varying this dimension directly determines the degree of spatial confinement of the asphalt molecules and changes the scale of the microscopic interaction network within the basic periodic unit. For example, due to the highly restricted spatial scale in the thinnest model (AS1), an asphaltene-phenol molecule has a limited coordination environment, allowing it to interact with a maximum of only two asphaltene-pyrrole molecules before encountering periodic constraints. In contrast, the extended (z)-dimension in the thickest model (AS4) provides a broader interaction space, allowing the same molecule to potentially interact with up to eight asphaltene-pyrroles. Therefore, progressively increasing this (z)-axis dimension effectively simulates the transition from a highly confined spatial state to a broader intermolecular interaction network.

### 2.2. Model Construction for Water Molecule Erosion

**Figure 3:**
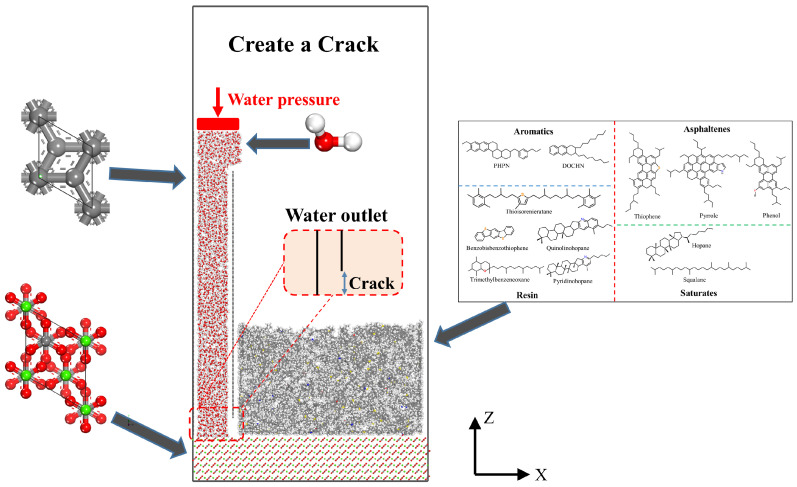
Model construction process.

In this study, MD simulations were employed to investigate the migration and erosion behavior of water molecules at the asphalt–aggregate interface. As shown in Figure 3, both the asphalt–aggregate–solution ternary interface model and the water migration model were constructed using Materials Studio. The detailed model construction procedure is described as follows:

(1) Crystal substrate and simulation box construction: A calcium carbonate (CaCO_3_) crystal slab was generated based on the {1 0 4} crystallographic plane and expanded along the U and V directions to form an aggregate substrate with dimensions of 110 Å × 30 Å × 20 Å (length × width × height). A vacuum region with a height of 160 Å was added above the aggregate surface, resulting in a simulation box with a total height of approximately 180 Å.
(2) Water and asphalt molecular models: Individual molecular models of water and asphalt were constructed using the Amorphous Cell module. For the water migration model, a specific number of 8000 water molecules was added to the simulation box based on a target density of 1.0 g/cm^3^. The initial density of the asphalt models was set according to the experimental value for the AAA-1 asphalt binder.
(3) Boundary condition setup: Two vertical rigid barriers were introduced at both ends of the simulation box to clearly define the flow boundaries of water molecules during the simulation.
(4) Ternary interface assembly (aggregate–asphalt–solution): The pre-optimized asphalt structure was placed on the aggregate crystal surface to ensure close interfacial contact. Water molecules were then added between the rigid barriers to complete the asphalt–aggregate–solution ternary interface model.
(5) Geometric optimization and initial dynamic equilibration: After construction of the interface model, geometric optimization was performed using the Smart algorithm to minimize the total potential energy. A preliminary dynamic equilibration simulation was then conducted for 1 ns under NVT ensemble conditions at 298.15 K using the Nose thermostat. This procedure was used to eliminate the nonequilibrium stress generated during the initial modeling process and to achieve preliminary dynamic stability.
(6) A water flow channel was introduced by truncating the bottom section of one of the vertical rigid barriers, creating a crack with a width of 10 Å. The specific width was governed by the size of the removed barrier segment. To initiate water migration, a downward vertical external force was subsequently applied, actively driving the water molecules through the artificial crack into the asphalt–crystal interface.

### 2.3. Tensile Simulation Procedure for the Asphalt Model

To further investigate the nanoscale tensile fracture mechanism of asphalt, secondary development based on MD simulations was carried out using a Perl script to implement continuous displacement-controlled loading of the asphalt molecular model.

**Figure 4:**
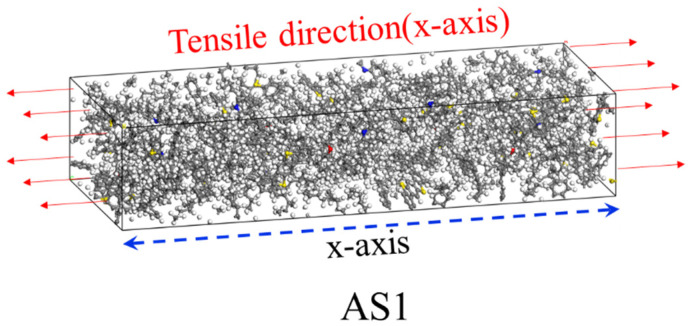
Schematic of asphalt tension.

(1) Geometric optimization: The asphalt molecular structure described in Section 2.1 was first optimized using a geometric optimization algorithm to eliminate unreasonable bond lengths, bond angles, and atomic overlaps in the model, and to ensure that the molecular structure reached a stable state with locally minimized energy.
(2) Equilibration phase: To ensure a reasonable initial configuration and avoid high-energy atomic overlaps during the random packing process, the initial density of the asphalt models was set at 0.5 g/cm^3^. The asphalt model was then equilibrated under the NPT ensemble. Following the method proposed by Luo [28,29,30], the model underwent annealing for 5 ns under the NPT ensemble, followed by an additional 1 ns NPT equilibrium simulation to obtain a stable asphalt structure. The minimum dimensions of the equilibrated asphalt simulation box are listed in Table 2. During the simulation, the temperature was controlled using the Nosé thermostat, and the pressure was controlled using the Andersen barostat.
(3) Tensile simulation: Considering both simulation accuracy and computational efficiency, a uniaxial tensile simulation was performed under the NVT ensemble at a loading rate of 0.01 Å/ps in the direction shown in Figure 4. To ensure the statistical reliability of the quantitative data, at least three independent simulation trials were conducted for each asphalt thickness model, using different initial atomic velocity distributions to account for stochastic effects.

## 3. Results and Discussion

### 3.1. Temperature and Density Evolution

**Figure 5:**
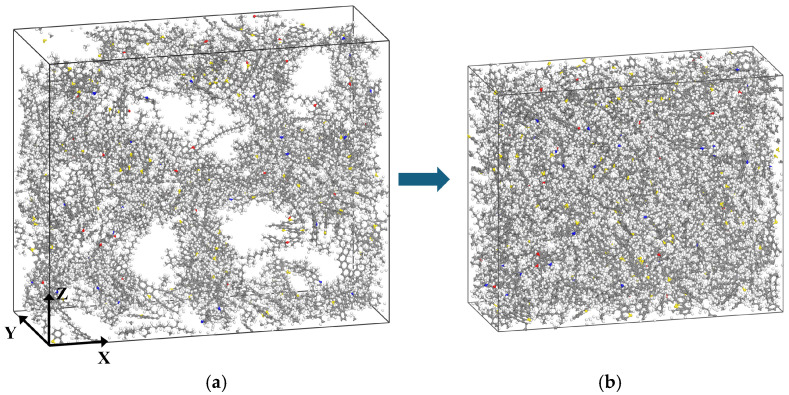
Snapshot of the asphalt model configuration: (**a**) before equilibration; (**b**) after equilibration.

Taking the AS4 asphalt as an example, Figure 5 displays the spatial configuration snapshots of the model before and after the equilibration phase.

**Figure 6:**
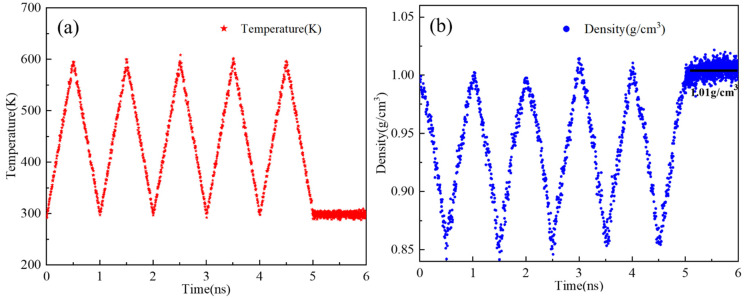
Thermodynamic changes during the annealing process: (**a**) Temperature change; (**b**) density change.

Figure 6 shows the temperature and density variations of asphalt models with different thicknesses during the annealing process at a heating rate of 6 K/10 ps. Annealing involves gradually increasing the temperature while allowing molecular relaxation, which promotes the rearrangement of asphalt molecular chains, relieves internal stress, and reduces structural defects, thereby optimizing the microstructure of the material.

The density of the system depends on its molecular composition and decreases with increasing temperature. Because AS1, AS2, AS3, and AS4 were all constructed using the same molecular composition ratios, their density profiles exhibited similar trends during annealing and ultimately stabilized at approximately 1.01 g/cm^3^. This result is in excellent agreement with the density values reported by Luo et al. [30]. The corresponding model dimensions at this equilibrium density are listed in Table 2.

### 3.2. Water Erosion at the Asphalt–Aggregate Interface

In MD simulations, non-bonded energy is commonly used to characterize non-covalent interactions within or between molecules, mainly including van der Waals interactions and electrostatic interactions. It effectively reflects the structural stability of a system, interfacial adhesion properties, and the interaction mechanisms between molecules. Depending on the research object and system composition, non-bonded energy can be classified into two types: internal non-bonded energy and inter-system non-bonded energy. For a single-material system, non-bonded energy generally refers to the non-bonded interactions among atoms or molecules within the system. For example, in a single asphalt system, non-bonded energy is used to characterize the interactions among asphalt components and the stability of the internal structure of the material. In contrast, for a composite system, such as an asphalt–aggregate system, both internal and inter-system non-bonded energies need to be considered. In this case, inter-system non-bonded energy specifically refers to the interfacial interactions between different material components. It is usually calculated by dividing the non-bonded interaction energy between asphalt and aggregate by the interfacial contact area and is defined as the interfacial adhesion energy ($W_{as-agg}$, mJ/m^2^), which is used to more objectively evaluate interfacial adhesion performance and strength. The internal non-bonded energy of the system refers to the non-bonded interaction energy among the components within the asphalt material itself. It is used to characterize molecular structural rearrangement and the response mechanism of the material under tension or other external loading conditions and is typically expressed in kcal/mol.

The non-bonded energy expression [30] is:

(1) $$E_{non-bond}(r)=E_{vdW}(r)+E_{elc}(r),$$

The electrostatic interaction term is expressed as:

(2) $$E_{elc}\;(r)=\frac{1}{4\pi \varepsilon_{0}}\cdot \frac{q_{i}q_{j}}{r},$$

The van der Waals interaction is expressed as:

(3) $$E_{vdW}(r)=4\varepsilon [{\left(\frac{\sigma }{r}\;\right)}^{12}-{\left(\frac{\sigma }{r}\;\right)}^{6}],$$

The interface adhesion energy is expressed as:

(4) $$W_{as-agg}=\frac{E_{non-bond}(as-agg)}{A},$$

where σ is the collision distance, r is the distance between particles, ε is the potential depth, $\varepsilon_{0}$ is the vacuum dielectric constant, $q_{i}$ and $q_{j}$ are the charges of the two charged particles, $E_{non-bond}(as-agg)$ is the non-bonded energy between asphalt and aggregate, and $W_{as-agg}$ is the adhesion energy between asphalt and aggregate.

**Figure 7:**
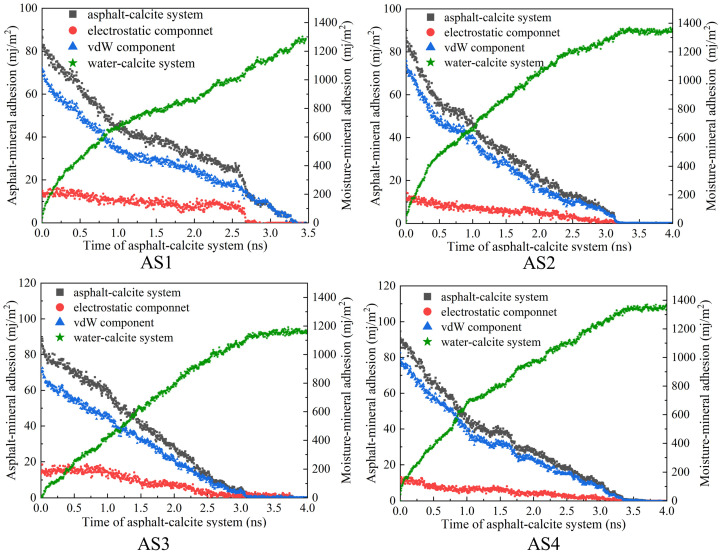
Evolution of adhesion energy between asphalt and aggregate during the water infiltration process.

The variation in interfacial adhesion energy between the Calcite {1 0 4} crystal surface and asphalt films of different thicknesses during water erosion was calculated. As shown in Figure 7, the interfacial adhesion energy increased with asphalt thickness. Further analysis revealed that the electrostatic interaction energy remained relatively stable as thickness increased. In contrast, the van der Waals (vdW) interaction energy showed a pronounced upward trend. These results indicate that the improved adhesion in thicker films is primarily attributed to the enhancement of interfacial vdW interactions.

Overall, asphalt films of various thicknesses exhibited similar trends during water intrusion. As water molecules entered the interface, the asphalt was displaced, reducing the contact area and causing a steady decrease in asphalt–Calcite adhesion energy, while the water–Calcite adhesion energy increased.

The point where asphalt–mineral adhesion drops to zero signifies that water molecules have completely displaced the asphalt film. This state represents total interfacial debonding. In the AS1 system, for instance, the adhesion energy reaches zero at approximately 3.3 ns, marking the completion of the stripping process. Beyond this threshold, the system enters a stable post-debonding phase. In this phase, energy components plateau and remain constant, a trend clearly evidenced by the AS2 system after 3.0 ns. This stabilization confirms that the simulation duration is sufficient to capture the entire evolution from initial moisture damage to ultimate adhesion failure. These results align with the experimental findings by Hou et al. [31], which demonstrated that moisture reduces the effective contact area and leads to adhesion failure.

**Figure 8:**
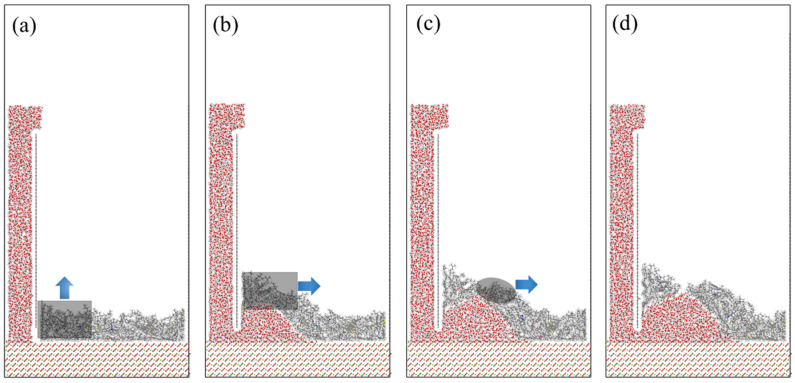
Water molecule piercing the AS1 asphalt process.

During this process, clear differences emerged among asphalt films with different thicknesses. As shown in Figure 8, the AS1 model exhibited significant deformation and eventually fractured. The AS2, AS3, and AS4 models also showed different degrees of deformation, with AS2 being more severely affected, whereas AS3 and AS4 exhibited comparatively milder responses. These observations indicate that increasing asphalt thickness significantly enhances resistance to moisture-induced damage.

Figure 8 shows the fracture process observed in the AS1 model. After water molecules began to penetrate the asphalt–aggregate interface, the asphalt was gradually squeezed toward the left side, forming a noticeable bulge. This bulge intensified over time and eventually resulted in material fracture. This simulated behavior is consistent with previous experimental observations [32,33], which confirmed that localized deformation caused by water can puncture asphalt and create pathways for further moisture infiltration.

Figure 8 also illustrates the fracture process of asphalt under the action of water molecules. The moisture-induced degradation process can be divided into two distinct stages. In Figure 8a,b, water molecules began to infiltrate the asphalt–aggregate interface and gradually lifted part of the asphalt; however, no obvious fracture was observed at this stage. As water molecules continued to penetrate deeper into the interface, a wedge effect gradually developed, as shown in Figure 8c. The increasing wedging pressure generated significant lateral forces in the interfacial region, resulting in localized stress concentration. Driven by these lateral forces, cracks initiated in mechanically weak regions of the asphalt and ultimately caused material failure.

These observations indicate that asphalt thickness is a critical factor affecting interfacial durability and resistance to moisture-induced damage. However, insights obtained solely from moisture intrusion simulations are insufficient to fully elucidate how asphalt thickness influences tensile fracture behavior at the interface. To address this limitation, MD simulations were further employed to investigate the nanoscale tensile fracture behavior of asphalt with different thicknesses. The aim was to clarify how thickness affects the microscopic fracture patterns of asphalt under uniaxial tension.

Compared with conventional experimental and numerical methods, MD simulations provide a more detailed description of component migration during crack formation. This enables a clearer understanding of the relationship between molecular migration and crack initiation and propagation.

### 3.3. Thickness Effect on Nanoscale Asphalt Cracking

#### 3.3.1. Morphological Evolution of Nanoscale Cracks

**Figure 9:**
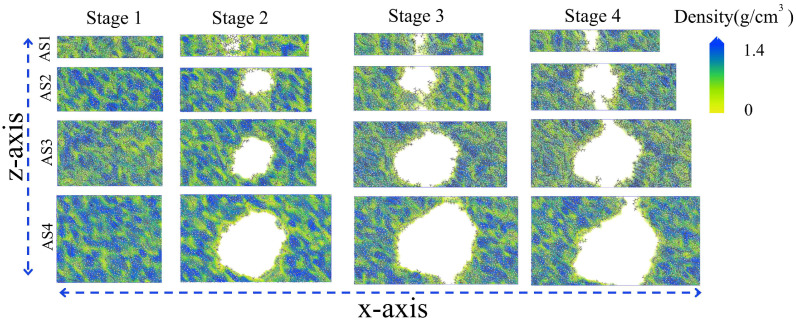
Tensile cracking process of asphalt with different thicknesses.

Figure 9 illustrates the morphological evolution of asphalt during the tensile process. As tensile loading is applied, the asphalt structure gradually elongates along the x-direction. Owing to the viscoelastic nature of asphalt, the molecular chains undergo significant deformation and migration under external stress. In this figure, we observe pronounced variations in internal molecular density as tensile deformation progresses. In the early stage (Stage 1), only minor structural adjustments and a slight weakening of intermolecular interactions occur.

As the tensile process continues (Stage 2), the increased dimension in the x-direction provides greater freedom for molecular movement, resulting in a distinct redistribution of local number densities. Structurally weak zones begin to emerge where the molecular population is sparse. With continued tensile deformation (Stage 3 and 4), these weak zones expand until the local density eventually decreases to zero, leading to the formation and coalescence of nanovoids. Although AFM observes surface features, our bulk simulations qualitatively align with the chemical heterogeneity reported by Sultana et al. [8], where internal de-peptization acts as a precursor to surface failure.

The observed evolution of nanovoids and their coalescence into cracks in our AS1-AS4 models is consistent with the findings of Cui et al. [34], who reported that the local concentration of specific SARA fractions governs the volume and surface area expansion of nanovoids during tensile loading.

#### 3.3.2. Stress–Strain Response and Tensile Strength

**Figure 10:**
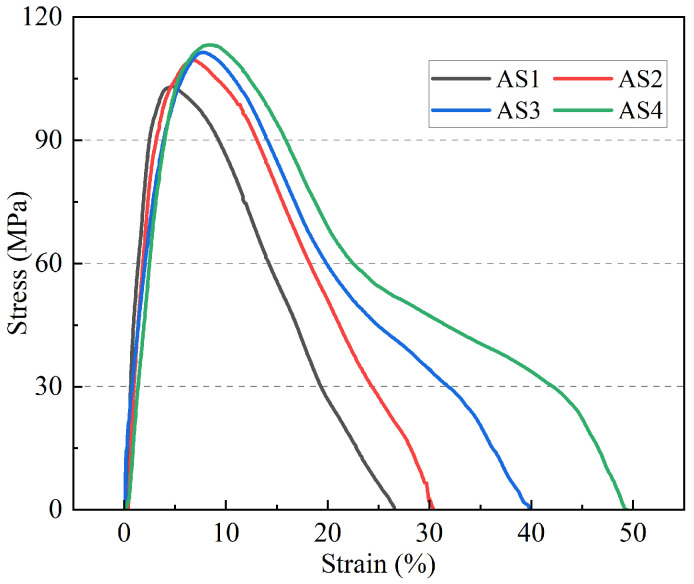
Stress–strain curves of asphalt models with different thicknesses.

As illustrated in the stress–strain curves(Figure 10), all models exhibit a characteristic viscoelastic response consisting of an initial near-linear elastic region, a stress peak, and a subsequent strain-softening stage. In the early loading phase (strain < 5%), the stress rises sharply as the molecular network resists deformation. The peak stress, representing the cohesive strength of the asphalt, shows a clear dependence on film thickness: the maximum stress increases from approximately 103.2 MPa for AS1 to 113.8 MPa for AS4. This enhancement in peak strength suggests that increased thickness facilitates a more reliable molecular entanglement network, providing higher resistance against initial structural separation.

The most pronounced effect of thickness is observed in the post-peak softening behavior and ultimate failure strain. After reaching the peak, the stress in the AS1 system drops rapidly, reaching complete failure at a strain of approximately 26.5%. In contrast, thicker models (AS3 and AS4) demonstrate a significantly more gradual stress decay with a prolonged tailing effect. Specifically, the failure strain of AS4 extends to approximately 49.6%, nearly double that of AS1. This increased ductility is associated with the slower coalescence of nanovoids in thicker films, which delays the formation of the final fracture surface.

#### 3.3.3. Energy Variation During the Tensile Process

**Figure 11:**
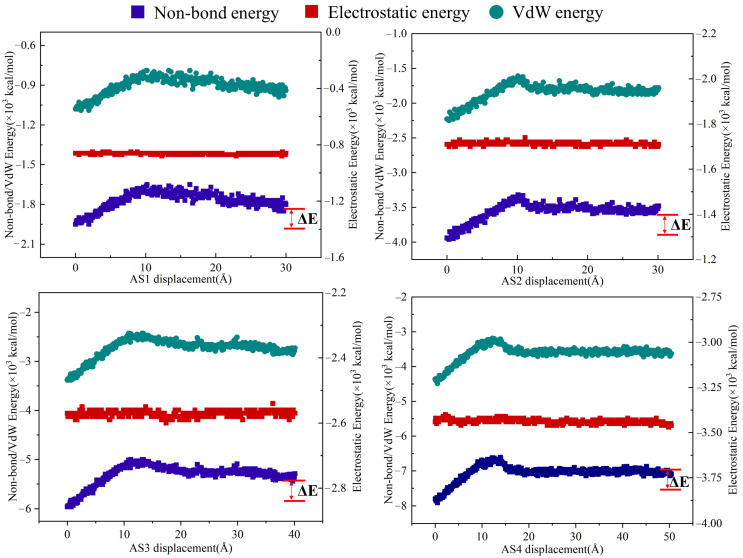
Energy changes of asphalt during tensile loading.

Figure 11 shows the variation in the internal non-bonded energy of asphalt during the tensile process, indicating that the energy introduced by tensile loading is primarily stored in the form of non-bonded interactions. At the initial loading stage, the entangled molecular chains within the asphalt gradually loosen and begin to rearrange, marking the onset of the linear elastic stage. During this stage, the internal non-bonded energy increases progressively with the applied load. As the external displacement continues to increase, the entangled network of molecular chains is progressively disrupted, leading to further relaxation and rearrangement of the molecular structure. At this stage, the system enters the plastic deformation phase, during which a slight decrease in internal non-bonded energy is observed. This behavior suggests that the variation in internal non-bonded energy [29] (ΔE) can serve as an effective indicator of the energy required for asphalt fracture.

**Figure 12:**
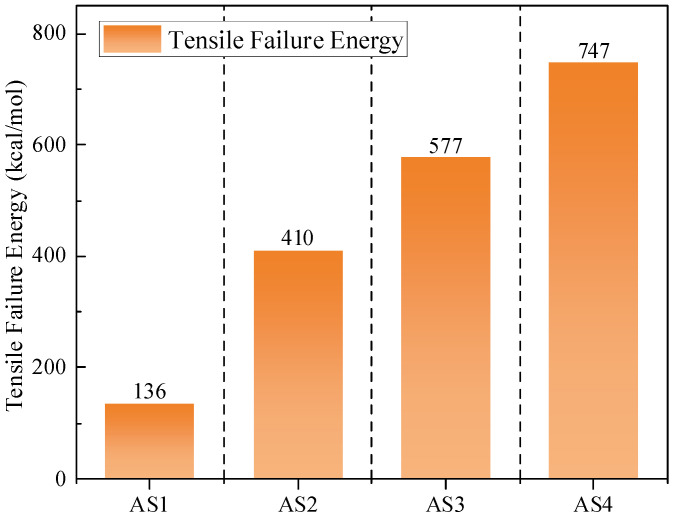
Tensile failure energy of asphalt with different thicknesses.

The internal non-bonded energy (ΔE) variation reflects the dynamic processes of molecular loosening and crack propagation. The area under the stress–strain curve represents the external work required for fracture, which is primarily stored in the form of non-bonded interactions. As shown in Figure 12, the energy required for tensile failure increases significantly with asphalt thickness, rising from 136 kcal/mol for AS1 to 747 kcal/mol for AS4. This trend indicates that asphalt thickness has a pronounced effect on resistance to tensile failure.

For asphalt systems with different thicknesses, the variation in internal non-bonded energy further reflects the dynamic processes of molecular loosening, rearrangement, and crack propagation. These energy variations directly affect the fracture resistance and durability of asphalt materials, providing a molecular-level interpretation of their mechanical behavior under tensile stress.

In thinner asphalt systems, the relatively small number of molecules results in a lower total non-bonded energy. During tensile deformation, the system must overcome a smaller potential energy barrier to disrupt intermolecular attractions. Consequently, molecular chains in thinner films rearrange with less resistance, making them more susceptible to crack propagation and leading to lower fracture energy.

In contrast, thicker asphalt systems possess a significantly larger population of molecular chains. This higher molecular density substantially increases the total non-bonded energy, which primarily consists of van der Waals and electrostatic interactions. During tensile failure, the work required to overcome these non-bonded potentials is much greater. Therefore, thicker asphalt undergoes a more pronounced plastic deformation stage. In this stage, a higher amount of energy is consumed to disrupt the extensive non-bonded interactions as nanopores and microcracks form and propagate.

These findings suggest that asphalt systems with greater thickness exhibit superior crack resistance because the increased energy dissipation during deformation contributes to improved durability under tensile stress.

### 3.4. Asphalt Component Evolution During Tensile Cracking

**Figure 13:**
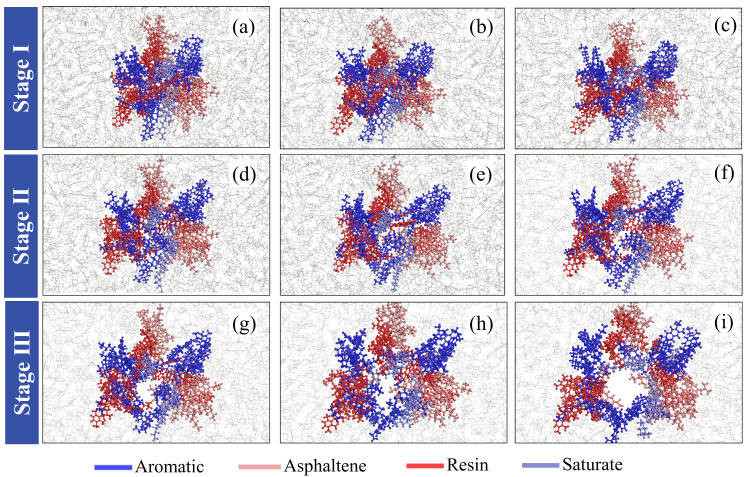
Morphological changes in the crack region of AS3 asphalt during the tensile process.

**Figure 14:**
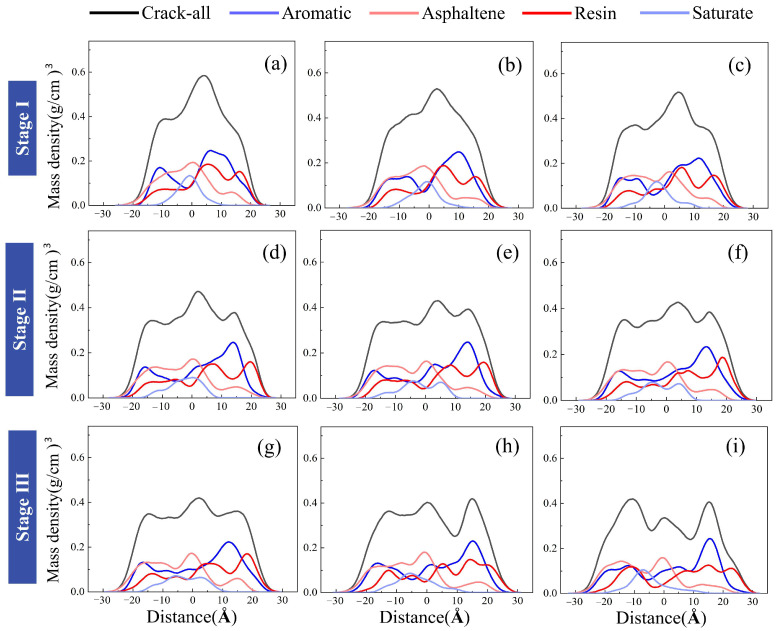
Component distribution characteristics of AS3 asphalt during the tensile process.

Figure 13 and Figure 14 take AS3 as an example to jointly illustrate the dynamic evolution of component concentrations in the crack region of asphalt during tensile loading. In Figure 14, the crack centerline is defined as the coordinate origin, and the concentration variations of each component during the tensile process are presented visually.

In Stage I (Initial Tensile Deformation and Relaxation), the asphalt exhibits a distinct high-peak concentration distribution in the crack-center region, as shown in Figure 14a. This indicates a high density of molecules near the incipient crack site. Although individual components (aromatics, asphaltenes, resins, and saturates) show different distribution patterns, their relative positions remain generally stable while the cluster structure gradually relaxes (Figure 13a–c). Physically, this stage is characterized by the diffusion of aromatics, whose concentration peak becomes lower and broader as they migrate toward both sides of the crack. This migration represents a molecular-level stress-relaxation mechanism; due to their relatively low molecular weight and non-polar nature, aromatic molecules—acting as the solvent phase—preferentially redistribute to accommodate volumetric expansion and alleviate local stress concentrations [35]. Simultaneously, the concentration of asphaltenes begins to shift, indicating the onset of structural adjustment under external load.

In Stage II (Micellar Destabilization and Disintegration), as tensile deformation continues, the asphalt concentration at the crack center further decreases. A critical observation in this stage is the significant shift of the resin concentration centroid toward the right side (Figure 14d), involving specific molecules such as trimethylbenzeneoxane and benzobisbenzothiophene. Resins act as peptizing agents to stabilize asphaltene clusters, reducing the polarity gradient between asphaltenes and the maltene phase [34,35]. The pronounced shift of resins observed in our simulation indicates a de-peptization process, where the destabilized asphaltene micelles lose their protective shells. This structural evolution, consistent with the morphology in Figure 13, directly reduces the stability of the asphalt clusters, leading to their subsequent disintegration. The “de-peptization” process identified in Stage II of our study, where resins migrate away from asphaltene clusters, aligns with the colloidal theory supported by Liu et al. [36], which identifies resins as the critical stabilizing barrier for asphaltene micelles.

In Stage III (Fracture Propagation and Re-aggregation), the crack width increases further, resulting in the total disintegration of the original asphalt clusters and a substantial rearrangement of all component concentrations. As shown in Figure 14i, resins and asphaltenes reach new concentration peaks (e.g., at −15 Å and 0 Å), which is consistent with the morphology in Figure 13i. This stage reveals that new cluster structures have formed, reassembling specifically around polar components. This re-aggregation of polar components is driven by the thermodynamic tendency to minimize surface free energy through the formation of new stable interfaces after the disruption of the initial bulk structure.

In summary, the tensile process of AS3 asphalt involves both molecular migration and structural collapse. Specifically, Stage I involves overall molecular diffusion; Stage II entails resin-driven de-peptization; and Stage III comprises the re-aggregation of polar components.

### 3.5. Crack Initiation in Asphalt

Common parameters, such as the Gaestel Index and the Asphaltene Index, are widely used to evaluate the instability of the asphalt colloidal structure [27,37,38]. Here, I_C_ is the Gastel Index, I_A_ is the Asphaltene Index, AS is the Asphaltene content, SA is the Saturates content, AR is the Aromatics content, and RE is the Resin content.

(5) $$I_{c}=\frac{AS+SA}{RE+AR},$$

(6) $$I_{A}=\frac{AS+RE}{SA+AR},$$

There remains some controversy regarding the influence of asphalt binder composition and structure on fracture and fatigue performance. Wang et al. [39] reported that the fatigue life of asphalt binder gradually decreases with increasing asphaltene content, attributing this primarily to the strong polarity of asphaltenes, which enhances intermolecular interactions but potentially increases brittleness. In contrast, Salehfard et al. [40] suggested that fatigue life increases with asphaltene content, proposing that a higher asphaltene concentration promotes a bulk effect that helps inhibit microcrack propagation. This study aims to resolve these conflicting observations by introducing the Asphaltene Index (I_A_) to quantitatively link localized chemical composition to the mechanical failure observed at the nanoscale.

Physically, I_A_ serves as a descriptor of the polar skeleton density within the asphalt matrix. In asphalt, asphaltenes and resins exhibit relatively strong polarity, whereas aromatics and saturates exhibit relatively weak polarity, forming a compositionally heterogeneous structure. As established in the analysis of Section 3.4, the stability of this asphaltic micellar structure is primarily maintained by resins acting as peptizing agents, which mitigate the polarity gradient between asphaltene clusters and the maltene phase.

**Figure 15:**
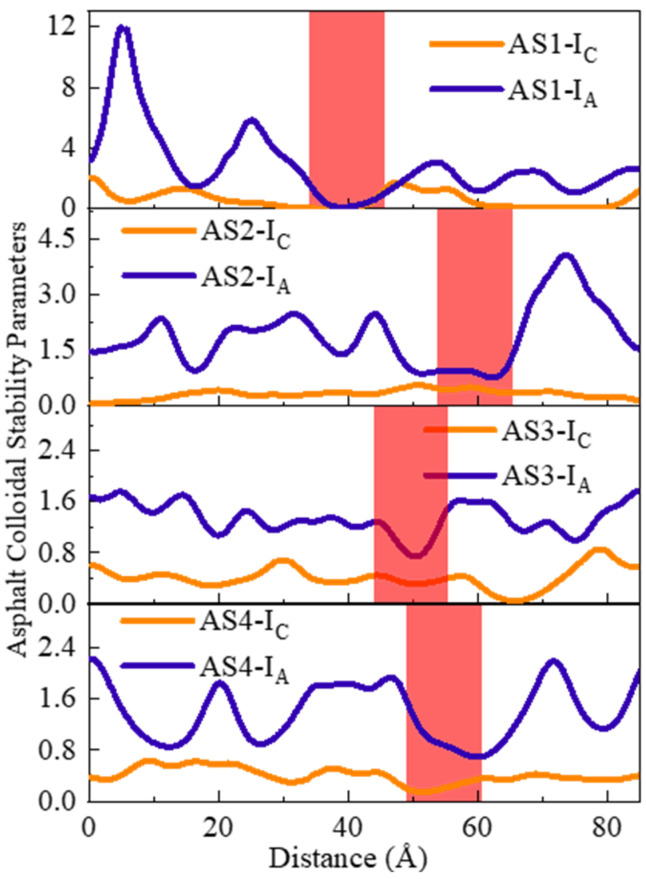
Distribution of I_A_ and I_C_ on the x-axis before tension.

Figure 15 displays the distribution of I_A_, where the red regions indicate the locations of initial crack formation. A significant spatial correlation is observed: crack initiation sites in all models (AS1–AS4) consistently correspond to regions with the minimum I_A_ values. This correlation was consistent across all three independent simulation trials for each thickness, confirming that I_A_ is a statistically valid indicator for identifying mechanical weak points. The observed correlation is linked to the de-peptization process in Stage II. Regions with low I_A_ values have lower concentrations of asphaltenes and resins, making them highly non-polar. As a result, they lack the necessary intermolecular interactions to support external loads. Without enough stabilizing resins, the micellar structure becomes unstable and yields under stress. This agrees with Sun et al. [41,42], who demonstrated that regions lacking polar asphaltene molecules have lower cohesive energy and act as structural weak points.

Under tensile stress, these weak links cannot effectively dissipate energy through complex molecular rearrangements, leading to the formation of nanovoids and subsequent cavitation. In contrast, high I_A_ regions, characterized by dense dipole-dipole interactions and strong cohesive bonds, provide superior resistance to crack propagation. Therefore, I_A_ serves as a predictive indicator of the localized failure trajectory, indicating that crack initiation in MD simulations depends on the colloidal stability of the binder at the molecular level. The I_A_ index connects bulk stability with surface degradation, as internal molecular rearrangements eventually cause the topographic changes detected by AFM. This provides a mechanistic basis for comparing nanoscale simulation results with microscale experimental observations.

## 4. Conclusions

This study systematically investigated the influence of asphalt thickness on nanoscale cracking behavior and component migration. The main conclusions are summarized as follows:

(1) Quantitative Mechanical Response: Increasing asphalt thickness significantly enhances tensile resistance. As the film thickness increases from AS1 to AS4, the peak stress rises from 103.2 MPa to 113.8 MPa, and the ultimate failure strain extends from 26.5% to 49.6%. Correspondingly, the fracture energy (ΔE) increases from 136 kcal/mol to 747 kcal/mol. This quantification provides a physical basis for understanding how the volume of the polar skeleton governs energy dissipation.
(2) Three-Stage Migration Mechanism: The tensile process is defined by a dynamic transition of the micellar structure: (i) structural relaxation and aromatics-driven stress relief; (ii) resin-driven de-peptization, leading to the disintegration of asphaltene clusters; and (iii) polar component re-aggregation to minimize surface free energy.
(3) Predictive Indicator for Failure: The Asphaltene Index (I_A_) is a reliable predictive indicator for identifying mechanical weak links. Cracks consistently initiate in regions with minimum I_A_ values, where insufficient polar reinforcement leads to micellar instability and nanovoid formation.

While restricted to nanoscale systems and accelerated loading rates (0.01 Å/ps) due to computational constraints, this study offers valuable molecular-level insights into asphalt fracture mechanisms. Future research will utilize multi-scale simulations to incorporate environmental stressors, such as oxidative aging, to better predict long-term pavement fatigue life. Despite these limitations, this work links localized chemical heterogeneity and macroscopic fracture resistance. By establishing the I_A_ index as a quantitative descriptor, this study clarifies the molecular mechanisms by which chemical composition and film thickness influence pavement durability, while providing mechanistic insights into the surface failures observed in AFM studies.

## Figures and Tables

**Table 1:** Number of molecules and mass fraction of the 12 components of asphalt model.

Components	Molecular Composition	Molecular Formula	Simplest Ratio	Mass Fraction (%)
Asphaltenes	Asphaltene-phenol	C_42_H_46_O_5_	3	5.3
	Asphaltene-pyrrole	C_66_H_67_NO_7_	2	5.5
	Asphaltene-thiophene	C_51_H_54_O_5_S	3	6.5
Resins	Benzobisbenzothiophene	C_18_H_10_O_2_S_2_	5	13.4
	Pyridinohopane	C_36_H_53_NO_2_	4	6.2
	Quinolinohopane	C_40_H_55_NO_2_	4	6.8
	Thioisorenieratane	C_40_H_55_O_3_S	4	7.0
	Trimethylbenzeneoxane	C_29_H_48_O_2_	15	6.4
Saturates	Hopane	C_35_H_62_	4	5.9
	Squalane	C_30_H_62_	4	5.2
Aromatics	DOCHN	C_30_H_42_O_2_	13	16.2
	PHPN	C_30_H_36_O_4_	11	15.7

**Table 2:** Model sizes of asphalt with different thicknesses after stabilization.

Asphalt Thickness	x-Axis	y-Axis	z-Axis
AS1	87.02	34.48	18.03
AS2	86.84	34.40	35.98
AS3	86.71	34.35	53.89
AS4	86.78	34.38	71.94

## Data Availability

The original contributions presented in this study are included in the article. Further inquiries can be directed to the corresponding author.

## Abbreviations

The following abbreviations are used in this manuscript:

SCB	Semi-circular bending test
DIC	Digital image correlation technique
FEA	Finite element analysis
XFEM	Extended finite element method
CZM	Cohesive zone model
DEM	Discrete element method
AFM	Atomic force microscopy
MD	Molecular dynamics
AS	Asphaltene content
SA	Saturates content,
AR	Aromatics content
RE	Resin content
*ΔE*	Internal non-bond energy
*I_C_*	Gastel Index,
*I_A_*	Asphaltene Index,
*E_non-bond(as-agg)_*	Non-bonded energy between asphalt and aggregate
*W_as-agg_*	Adhesion energy between asphalt and aggregate
